# Supplementary material for: Anti-HIV Humoral Response Induced by Different Anti-Idiotype Antibody Formats: An In Silico and In Vivo Approach
Source: Int J Mol Sci. 2024 May 24;25(11):5737. doi: 10.3390/ijms25115737 (PMC11171986; doi:10.3390/ijms25115737)
Supplement: Supplementary file 1 [file ijms-25-05737-s001.zip › ijms-2942453-supplementary.pdf]

International Journal of *Molecular Sciences*

MDPI

Supplementary tables

Supplementary Table S1. *Linear epitope of the different constructs defined by immunoinformatic tools.*

	ScFVHVL	ScFVLVH	Mb VHVL	Mb VLVH
**B cell Epitope**	**39-42**<br>GKSL	**169-171**<br>GKS	**39-41**<br>GKS	**170-171**<br>KS
	**98-153**<br>YGEDPFAYWGQ<br>GTLVTVSTAGST<br>SGSGKPGSGEGS<br>TKGELVMTQSPA<br>SLAVSLGQR	**5-18**<br>TQSPASLAVSLGQ<br>R	**98-151**<br>YGEDPFAYWGQ<br>GTLVTVSTAGST<br>SGSGKPGSGEGS<br>TKGELVMTQSPA<br>SLAVSLG	**6-17**<br>QSPASLAVSLGQ<br>R
	**166**<br>T	**31**<br>T	**164-167**<br>VSTS	
	**179-182**<br>PGQP	**44-47**<br>PGQP	**180-181**<br>GQ	**44-46**<br>PGQ
	**192-197**<br>NLESGV	**57-62**<br>NLESGV	**192-197**<br>NLESGV	**58-62**<br>LESGV
	**217-220**<br>VEEE	**82-85**<br>VEEE	**217-222**<br>VEEEDT	82-85<br>VEEE
	**231-236**<br>WEIPYT	**97-101**<br>EIPYT		
	**4-14**<br>SGAELVKPGAS	**107-143**<br>KLEIKRGSTSGSGK<br>PGSGEGSTKGLEQ<br>SGAELVKPGA	**4-13**<br>SGAELVKPGA	**103-143**<br>GGGTKLEIKRGS<br>TSGSGKPGSGEG<br>STKGLEQSGAEL<br>VKPGA
		**156**<br>F	**292**<br>F	
	**51-60**<br>NSGTTGYNQK	**181-191**<br>NSGTTGYNQKF	**51-60**<br>NSGTTGYNQK	**181-192**<br>NSGTTGYNQKF<br>K
	**72-73**<br>DQ	**200-203**<br>DQSS	**70-73**<br>DQSS	**198-203**<br>TVDQSS
	**83-86**<br>LTSE	**213-215**<br>LTS	**83-86**<br>LTSE	**214-215**<br>TS

	**72-73**<br>SS			
		**227-232**<br>YYGEDP		**228-232**<br>YGEDP
			**232-264**<br>EIPYTFGGGTKLE<br>IKREPKSPKSADK<br>THTAPPA	
			**306-322**<br>QPAENYKNTQPI<br>MDTDG	**304-323**<br>NGQPAENYKNT<br>QPIMDTDGS
			**335-342**<br>SNWEAGNT	**334-342**<br>KSNWEAGNT
			**351-360**<br>GLHNHHTEKS	**352-360**<br>LHNHHTEKS
			**271-280**<br>IPPPLEQMAK	**236-280**<br>WGQGTLVTVST<br>AEPKSPKSADKT<br>HTAPPAAPQVY<br>TIPPPLEQMAK
			**294**<br>P	**292-295**<br>FFPE

Supplementary Table S2. *Correlation between the epitope recognized by B cell and the subdivision in frameworks (ƒFrw) and complemetary-determinating regions (CDR) of the variable regions of our constructs.*

The table reports the correlation between the epitope recognized by B cell and the subdivision in frameworks (ƒFrw) and complemetary-determinating regions (CDR) of the variable regions of our constructs. When the epitope enclosed both CDR and ƒFrw, color-code is used to identify the different portions.

eEpitope	***VH***	***VL***
GKSL	*Frw2*	
YGEDPFAYWGQGTLVTVSTAGSTSGSGKPGSGEGSTKGELVMTQSPASL AVSLGQR	*CDR3 - Frw4*	*Frw1*
VSTS		*CDR1*

PGQP		*Frw2*
NLESGV		*Frw3*
VEEEDT		*Frw3*
WEIPYT		*CDR3*
GGGTKLEIKRGSTSGSGKPGSGEGSTKGLEQSGAELVKPGAS	*Frw1*	*CDR3-ƒFrw4*
NSGTTGYNQKFK	*Frw3*	
TVDQSS	*Frw3*	
LTSE	*Frw3*	
YYGEDP	*CDR3*	
EIPYTFGGGTKLEIKREPKSPKSADKTHTAPPA		*CDR3-ƒFrw4*

The table reports the correlation between the epitope recognized by B cell and the subdivision in frameworks (ƒFrw) and complemetary-determinating regions (CDR) of the variable regions of our constructs. When the epitope enclosed both CDR and ƒFrw, color-code is used to identify the different portions.
